# Implementing work-related Mental-health guidelines in general PRacticE (IMPRovE): findings of a parallel cluster randomised controlled trial

**DOI:** 10.1136/bmjment-2024-301330

**Published:** 2025-07-28

**Authors:** Danielle Mazza, Vera Camões-Costa, Karen Nolidin, Samantha Chakraborty, Justin Kenardy, Bianca Brijnath, Duncan Mortimer, Joanne Enticott, Michael Kidd, Lyndal J Trevena, Sharon Reid, Alex Collie

**Affiliations:** 1 Department of General Practice, School of Public Health and Preventive Medicine, Monash University, Melbourne, Victoria, Australia; 2 Cochrane Australia and the Australian Living Evidence Collaboration, School of Public Health and Preventive Medicine, Monash University, Melbourne, Victoria, Australia; 3 The University of Queensland, Brisbane, Queensland, Australia; 4 School of Humanities and Social Sciences, La Trobe University, Melbourne, Victoria, Australia; 5 School of Population and Global Health, The University of Melbourne, Melbourne, Victoria, Australia; 6 Centre for Health Economics, Monash University Faculty of Business and Economics, Clayton, Victoria, Australia; 7 Monash Centre for Health Research and Implementation, Monash University, Clayton, Victoria, Australia; 8 Centre for Future Health Systems, University of New South Wales, Sydney, New South Wales, Australia; 9 Nuffield Department for Primary Care Health Sciences, University of Oxford, Oxford, UK; 10 Sydney School of Public Health, University of Sydney, Sydney, New South Wales, Australia; 11 Speciality of Addiction Medicine, Central Clinical School, Faculty of Medicine and Health, Tha\e University of Sydney, Sydney, New South Wales, Australia; 12 Healthy Working Lives Research Group, School of Public Health and Preventive Medicine, Monash University, Melbourne, Victoria, Australia

**Keywords:** Depression & mood disorders

## Abstract

**Background:**

Mental health conditions arising from work are a rapidly increasing burden for individuals, employers and society, and are challenging to diagnose and treat.

**Objective:**

To assess the effectiveness of a multicomponent intervention on increasing general practitioners’ (GPs’) adherence to the ‘Clinical guideline for the diagnosis and management of work-related mental-health conditions in general practice’ (the Guideline) and improve patient work and health and work outcomes.

**Methods:**

Pragmatic hybrid III parallel cluster randomised controlled trial involving Australian GPs and their patients. GP clinics were randomly assigned to receive the intervention (GP participation in an academic detailing session, enrolment into a virtual community of practice, and receipt of resources). Those assigned to the control group received no support related to the implementation of the Guideline. GP adherence to guideline recommendations was assessed at baseline and 9 months postbaseline, using virtual simulated patient scenarios (vignettes) describing a diverse range of patient circumstances. Patient work and health outcomes (using the 21-item Depression and Anxiety Stress Scale and 36-item short-form) were assessed using self-report surveys.

**Findings:**

Thirty-eight intervention clusters (52 GPs) and 36 control clusters (46 GPs) contributed to the primary outcome data. Intervention clusters had significantly higher adherence scores than control clusters, by 0.98 points on a 0–9 scale (95% CI 0.38 to 1.58) with a Cohen’s d of 0.67. Patients recruited from 30 intervention (n=99) and 17 control (n=55) clusters contributed to the secondary outcome data. No differences were detected for patients’ work or health outcomes due to an underpowered sample.

**Conclusions:**

GP adherence to the Guideline improved as a result of receiving the multicomponent intervention.

**Implications:**

Purposively designed multicomponent implementation strategies to increase guideline-concordant care should be incorporated into guideline production activities and operationalised with guideline release to facilitate evidence-based care.

**Trial Registration number:**

ACTRN12620001163998, November 2020

**WHAT IS ALREADY KNOWN ON THIS TOPIC**

**WHAT THIS STUDY ADDS**

- An intervention which combined tailoring key messages (academic detailing), peer-to-peer support (academic detailing and engagement in a virtual community of practice (VCoP)), and a flexible learning environment (VCoP and resources) significantly improved adherence to the clinical guideline for the diagnosis and management of WRMHCs in general practice.

**HOW THIS STUDY MIGHT AFFECT RESEARCH, PRACTICE OR POLICY**

- Guideline release to facilitate evidence-based care should incorporate guideline implementation activities.

## Introduction

 Worldwide, work-related mental health conditions (WRMHCs) are a major and growing cause of disability, absenteeism, unemployment, early exit from the workforce, and lower lifetime earnings and income.^1^ These disorders incur substantial costs for workers, employers, and the whole of society and, in many countries, are the most rapidly rising cause of early exit from the workforce onto disability pension.^2^

In Australia, since 2000, the median time off work for workers with accepted WRMHC claims rose by 175%, from 11.2 working weeks in 2000–2001 to 30.8 weeks in 2019–2020.^3^ During the last two decades, the number of claims for WRMHCs with more than 5 days off work grew by 73%, and represented 28% of all disease claims in 2019–2020.^3^ Additionally, the median compensation per WRMHC claim increase during this period more than doubled in real terms over the period 2000–2001 to 2019–2020.^3 4^ Therefore, better management of WRMHCs may decrease the burdens incident on the patient, general practitioners (GPs), the health system, the compensation system and society at large.

In 2019, the ‘Clinical guideline for the diagnosis and management of work-related mental-health conditions in general practice’ (the Guideline) was published to support Australian GPs in their practice by providing evidence-based recommendations addressing 10 key clinical challenges associated with WRMHCs.^5^

However, the creation of clinical guidelines does not guarantee their implementation in practice.^6^ Frequently used passive approaches such as mailing education materials are unlikely to result in behaviour change when used on their own.^7^ Instead, complex multicomponent interventions actively targeting barriers to change are more likely to be effective.^7^ Academic detailing (involving peer-to-peer delivery of evidence-based key messages that are tailored to the needs of participating clinicians) and communities of practice are each successful at facilitating guideline implementation in general practice,^7 8^ and enhanced when offered together.^7 9^ The provision of resources to support guideline implementation is also more effective when combined with peer-to-peer strategies.^10^ The Implementing work-related Mental-health guidelines in general Practice (IMPRovE) Trial therefore sought to assess the effectiveness of a complex intervention involving these three strategies on GP adherence to the evidence-based guideline for the diagnosis and management of WRMHCs in general practice and on patient work and health outcomes.^11^

## Methods

### Trial design

IMPRovE was a parallel two-armed, pragmatic cluster randomised controlled trial. The full study protocol including rationale, design and methods has been previously published.^11^

### Recruitment and participants

GPs across Australia who could recruit 7–24 employed adult patients with a confirmed or suspected WRMHC prospectively over 12 months were eligible. Informed consent was obtained from 127 GPs from 96 general practice clinics (clusters; response rate 1.9%).

Patients were eligible if they were 18 years of age or older, had a confirmed or suspected WRMHC, were receiving care from a GP participant, and were employed (including on paid or unpaid leave) at the time of enrolment.

The recruitment period, for both GPs and patients, corresponded with the COVID-19 pandemic.

### Interventions

Participating GPs in clinics assigned to the control group were considered as undertaking usual practice, as they received no support related to the implementation of the Guideline. The intervention was delivered to GPs who were randomised to the intervention arm after collection of their baseline data.

The IMPRovE intervention consisted of active and passive components.^12^ The three intervention components (ie, a single 60 min online academic detailing session, enrolment and participation in a virtual community of practice (vCoP), and provision of hard-copy and electronic relevant resources) are described in more detail in online supplemental file 1. Academic detailing and engagement on the CoP (active components) were supplemented with the provision of resources (passive component) addressing clinical concerns, system issues and patient concerns. These approaches were introduced to GPs in a staged manner to facilitate guideline-concordant practice over time.^13^

### Randomisation and masking

After collection of baseline data, a statistician masked to the identity of the participants allocated GP clinics (or clusters) to the intervention or control arm with a 1:1 allocation ratio. GPs from the same clinic were grouped into the same cluster, with a minimum of one GP per cluster. Randomisation was performed as stratified randomisation using a minimisation procedure.^11 14^ Patients were allocated to the control or intervention arm based on the randomisation of their GP.

### Outcomes

The primary outcome, adherence to guideline recommendations by GP participants, was assessed at baseline and 9 months postbaseline, using virtual simulated patient scenarios (vignettes) describing a diverse range of patient circumstances. This approach has been used to evaluate practice and as an educational tool in compensable injury research with GPs in Europe^15^ and with physiotherapists in Australia.^16^

A set of 18 vignettes was developed to collectively represent the range of patient characteristics typically encountered by GPs when treating patients with WRMHCs and based on qualitative interviews with people with lived experience of WRMHCs (the method followed to develop this Guideline’s adherence tool will be published elsewhere). Each GP was allocated three randomly selected vignettes at baseline and another three vignettes at 9 months. Random allocation of vignettes to GPs was to ensure we evaluated adherence across a balanced representation of the full scope of clinical practice in both treatment and control groups. This approach sought to avoid learning effects, whereby an improvement in adherence might arise simply due to repeated exposure to the vignettes. Use of this tool required GPs to answer three questions per vignette, resulting in an adherence score ranging from 0.0 to 9.0 at each time point (by summing the responses to three presented vignettes).

To assess inter-rater reliability, two researchers independently scored the baseline vignettes (n=98), with a Cohen’s kappa of 0.92, which suggested near perfect agreement (kappa range for this classification: 0.81–0.99). Remaining discrepancies were reviewed by the two researchers, and 100% agreement was obtained. The same process was followed for the vignette responses completed at the 9-month follow-up.

Secondary outcome measures captured the effect of the intervention on patient health and work status. Patient health status was assessed using the 36-item short-form version 2 (SF-36 V.2)^17^ and the 21-item Depression and Anxiety Stress Scale (DASS-21).^18^ Work status was measured using the item ‘Are you currently working in a paid job?’ from the Australian National Return-to-work Survey.^19^ Patients could choose to complete surveys either electronically, on paper, or over the phone, at baseline, and at 3 months, 6 months and 9 months postbaseline.

### Statistical analyses

A description of the power considerations and statistical analysis used has been published in the protocol paper.^11^ For withdrawn participants, data were collected up until the point of withdrawal. No primary outcome data were requested to be excluded on withdrawal. Sensitivities of treatment effect estimate for missing outcome data were analysed using multiple imputation. All analyses were done with STATA (V.17). An independent data monitoring committee oversaw the study.^11^

### Patient and public involvement

The trial benefited from patient and public involvement through its governance structure comprised of a Steering Group, an Intervention Advisory Group and a GP Reference Group, each advising on different aspects of its design and conduct.

A steering group comprising members from key clinical organisations (representing Australian GPs, psychologists and psychiatrists) as well as representatives of compensation agencies and a key non-government mental health organisation provided oversight of the research, offered guidance about the health system and policy context informing intervention design, appropriateness, applicability and delivery for different jurisdictions, and ensured that the interventions aligned with concurrent and planned policy and regulatory initiatives.

The intervention was co-designed with GPs (as they were the end users of the intervention), and with policy makers who influence the compensation landscape. The intervention components were created in collaboration with a reference group of GPs, as well as an intervention advisory group that included GPs, mental health consumers, a representative from the Royal Australian College of General Practitioners, and representatives of workers’ compensation schemes. The Intervention Advisory Group provided advice to the team about the content and delivery of the vCoP. The GP Reference Group provided advice and input into the appropriateness of the intervention for general practice uptake, including commenting on the design, layout and content of the vCoP, piloting a test version of the vCoP, and reviewing educational materials and other content that were delivered through the vCoP.

## Results

**Figure 1:**
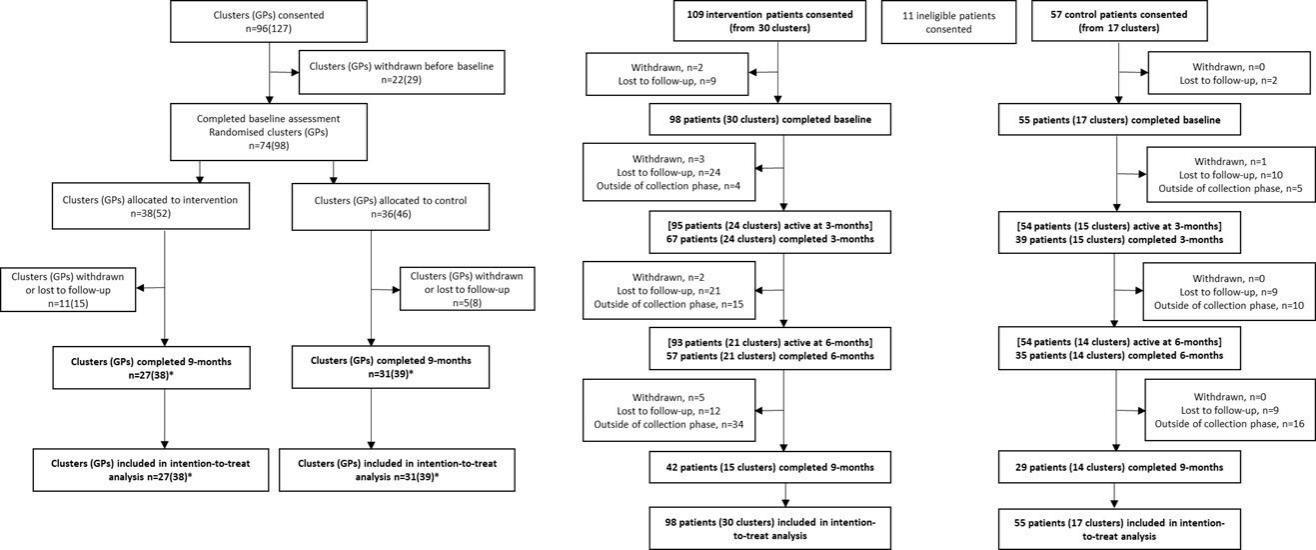
Flowchart for general practitioner (GP) and patient participation in each arm throughout the trial. ‘Intention-to-treat’ refers to change in adherence between baseline and 9 months. *includes one GP (in each group) who withdrew from the trial due to being unable to recruit patients but who agreed to complete 9-month data collection.

Recruitment of GPs across all states and territories in Australia, and GP data collection occurred from December 2020 to February 2022. Follow-up data were collected at 9 months postbaseline from September 2021 to December 2022. Figure 1 displays a flow chart of GP-participant numbers throughout the trial. In total, 74 clusters (98 GPs) completed full baseline data collection, while 58 clusters (77 GPs) returned full 9-month data.

Cohort characteristics at baseline are described in table 1. Both control and intervention GPs had, on average, 15 years of experience in practice, ranging from 1 years to 42 years for the control arm and 1.5 years to 44 years for the intervention arm. Most of the control and intervention GPs estimated that less than 50% of their patients had a workers’ compensation claim or review, with almost two-thirds of the control arm and half the intervention arm estimating they had only a small proportion of patients with a claim (<5%). Most control and intervention GPs had basic training related to mental healthcare, and approximately a third of the control arm and half the intervention arm were members of an online community.

**Table 1:** Baseline general practitioner (GP) and patient demographics and characteristics by arm

Characteristics	Control arm	Intervention arm
	General practitioners (n=46)	General practitioners (n=52)
Time in practice (years)	**16.1±11.7**	**14.8±9.9**
State of practice(s)		
Victoria	18 (39.1%)	23 (44.2%)
New South Wales	11 (23.9%)	17 (32.7%)
Queensland	10 (21.7%)	5 (9.6%)
South Australia	3 (6.5%)	3 (5.8%)
Western Australia	2 (4.3%)	1 (1.9%)
Australian Capital Territory	2 (4.3%)	1 (1.9%)
Northern Territory	0 (0.0%)	1 (1.9%)
Tasmania	0 (0.0%)	1 (1.9%)
Member of another online community	**16 (34.8%**)	**27 (51.9%**)
Estimated proportion of patients with workers’ compensation claim or review		
<5%	30 (65.2%)	23 (44.2%)
5% to <50%	11 (23.9%)	22 (42.3%)
50% to 100%	3 (6.5%)	7 (13.5%)
Don’t know	2 (4.3%)	0 (0.0%)
Qualifications relevant to compensable injury or mental health		
Mental health skills training level 1	40 (87.0%)	46 (88.5%)
Focused psychological strategies skills training level 2	11 (23.9%)	7 (13.5%)
Fellowship of the Australasian Faculty of Occupational and Environmental Medicine	2 (4.3%)	1 (1.9%)
Other*	2 (4.3%)	5 (9.6%)

Characteristics	Patients (n=98)	Patients (n=55)
Age in years (mean, SD, range)	49.76±8.76, Range: 32–69	47.44±11.28, Range: 26–68
Gender (n, %)		
Male	21 (38.18%)	29 (29.59%)
Female	34 (61.82%)	68 (69.39%)
Prefer not to say	0 (0.0%)	1 (1.02%)
State of clinic (n, %)		
Victoria	18 (32.73%)	39 (39.80%)
New South Wales	9 (16.36%)	30 (30.61%)
Queensland	12 (21.82%)	5 (5.10%)
South Australia	8 (14.55%)	10 (10.20%)
Australian Capital Territory	5 (9.09%)	7 (7.14%)
Western Australia	3 (5.45%)	0 (0.0%)
Northern Territory	0 (0.0%)	1 (1.02%)
Tasmania	0 (0.0%)	6 (6.12%)
Workers’ compensation claim in the past 2 years (n, %)		
Yes, the claim was successful	17 (30.91%)	28 (28.57%)
Yes, however the claim was not accepted	0 (0.0%)	2 (2.04%)
Yes, the claim is currently under review	5 (9.09%)	13 (13.27%)
No, I have not	29 (52.73%)	51 (52.04%)
I prefer not to say	3 (5.45%)	2 (2.04%)
I don't know	1 (1.82%)	2 (2.04%)

Data are mean (SD), or n (%).

* Includes Bachelor of Psychological Science (Honours), Anglican Counselling course, postgraduate Public Health degree, Master of Public Health (Occupational Medicine), Master of Psychopharmacology, Diploma or Postgraduate Diploma of Occupational Medicine.

Descriptive analyses revealed an increase in the mean adherence score over time for both the control and intervention arms (table 2). After adjusting for the stratification variables and the inclusion of cluster as a random effect, there was a significant increase of the adherence score from baseline to 9 months with the intervention compared with control by 0.98 (95% CI 0.38 to 1.58), with a Cohen’s d of 0.67. GP_ID was investigated as a random effect in the model to account for repeated measures, and the likelihood ratio test results showed that there was no benefit to having it included as a random effect (χ^2^(2) = 1.96, p=0.376). Adding mental health training as a stratification factor did not affect the magnitude of change of the adherence score for the intervention compared with control, showing an increase by 0.96 (95% CI 0.35 to 1.58) with a Cohen’s d of 0.66. Due to missing primary outcome data (ie, 7 control arm GPs and 14 intervention arm GPs completed baseline data collection but not the 9-month follow-up due to either withdrawal or being lost to follow-up), a sensitivity analysis was conducted to check the robustness of the primary analysis by performing multiple imputation, showing similar results to the non-imputed analysis (online supplemental file 2).

**Table 2:** Unadjusted mean adherence scores for general practitioners (GPs) by arm and time point with SD

Arm	Time point	
	Baseline	9 months
Control		
Mean (SD)	5.13 (1.22)	5.94 (1.33)
Range	2.5–9.0	3.5–9.0
N	46	39
Intervention		
Mean (SD)	5.37 (1.46)	6.45 (1.76)
Range	1.5–8.5	3.0–9.0
N	52	38

Recruitment of 168 patients from participating clusters across all states and territories in Australia occurred from 1 January 2021 to 30 September 2022. If patients did not submit a survey at any time point after baseline, they were considered ‘active’ in the trial until the end of the data collection phase unless they requested to be withdrawn. There were 58 patients who completed a survey in all time points, with the remaining patients completing a survey for at least one time point. Figure 1 displays the patient-participant numbers throughout the trial. At each time point, a greater proportion of surveys was collected from intervention patients than from control patients.

The patients’ characteristics are described in table 1. The mean age of high 40s was similar for both control and intervention patients as was the gender distribution. The majority of patients were recruited from Victoria and New South Wales. More than half of the patient sample (54%) had not submitted a workers’ compensation claim in the past 2 years. Of those (42%) who reported submitting a claim, the majority (71%) specified that their claim was successful.

In our protocol,^11^ we identified the need for a target sample of 224 patients from both control and intervention clusters, to obtain 90% power for detecting medium effects (Cohen’s d=0.5) in secondary outcome measures between the two groups at 3 months. At 3 months, we had only obtained 30% and 17% of the target sample for the intervention arm and control arm patients, respectively.

Descriptive analyses indicated decreasing severity of mental health symptoms over time in both groups as measured by the DASS-21 (table 3).

**Table 3:** Mean unadjusted score for each subscale of the DASS-21 and SF-36, number and proportion of patients who were working in a paid job over time, from each group at each time point and arm

	DASS subscale	Control	Intervention
Baseline	Depression	20.04 (SD=11.30, range: 0–42)	20.00 (SD=9.32, range: 0–40)
	Anxiety	17.02 (SD=9.53, range: 2–42)	17.69 (SD=9.80, range: 0–38)
	Stress	24.40 (SD=8.39, range: 6–42)	24.06 (SD=8.53, range: 4–42)
3 months	Depression	16.10 (SD=12.07, range: 0–42)	15.67 (SD=11.26, range: 0–40)
	Anxiety	14.46 (SD=10.66, range: 0–36)	12.09 (SD=8.80, range: 0–40)
	Stress	20.51 (SD=10.60, range: 2–40)	18.54 (SD=9.82, range: 2–36)
6 months	Depression	16.29 (SD=11.94, range: 0–42)	14.56 (SD=11.08, range: 0–42)
	Anxiety	12.91 (SD=9.55, range: 0–36)	12.53 (SD=8.08, range: 0–32)
	Stress	20.46 (SD=9.49, range: 4–40)	18.53 (SD=8.94, range: 4–38)
9 months	Depression	15.52 (SD=12.88, range: 0–40)	12.86 (SD=9.95, range: 0–34)
	Anxiety	2.07 (SD=8.89, range: 0–28	10.86 (SD=9.50, range: 0–40)
	Stress	18.21 (SD=10.55, range: 0–40)	15.95 (SD=9.93, range: 0–38)

	SF-36 component	Control	Intervention
Baseline	Physical	44.06 (SD=10.28, range: 10–61)	42.62 (SD=10.68, range: 22–62)
	Mental	30.59 (SD=10.11, range: 8–56)	29.60 (SD=9.11, range: 15–53)
3 months	Physical	43.09 (SD=9.31, range: 9–60)	41.54 (SD=10.42, range: 22–64)
	Mental	33.29 (SD=11.75, range: 11–56)	35.42 (SD=12.36, range: 15–70
6 months	Physical	42.31 (SD=10.61, range: 11–64)	42.34 (SD=9.42, range: 24–58)
	Mental	35.13 (SD=11.00, range: 11–58)	37.19 (SD=11.46, range: 18–63)
9 months	Physical	42.94 (SD=10.81, range: 11–62)	43.04 (SD=9.40, range: 9–59)
	Mental	37.85 (SD=12.53, range: 7–63)	38.27 (SD=12.30, range: 12–63)

	Working status	Control	Intervention
Baseline	Yes (n, %)	36 (65.45%)	63 (64.29%)
	No (n, %)	18 (32.73%)	34 (34.69%)
	Don’t know/Can’t say (n, %)	0 (0.00%)	1 (1.02%)
	Refuse to say (n, %)	1 (1.82%)	0 (0.00%)
3 months	Yes (n, %)	28 (71.79%)	49 (73.13%)
	No (n, %)	9 (23.08%)	18 (26.87%)
	Don’t know/Can’t say (n, %)	1 (2.56%)	0 (0.00%)
	Refuse to say (n, %)	1 (2.56%)	0 (0.00%)
6 months	Yes (n, %)	27 (77.14%)	40 (70.18%)
	No (n, %)	7 (20.00%)	16 (28.07%)
	Don’t know/Can’t say (n, %)	1 (2.86%)	1 (1.75%)
	Refuse to say (n, %)	0 (0.00%)	0 (0.00%)
9 months	Yes (n, %)	20 (68.97%)	26 (61.90%)
	No (n, %)	9 (31.03%)	14 (33.33%)
	Don’t know/Can’t say (n, %)	0 (0.00%)	0 (0.00%)
	Refuse to say (n, %)	0 (0.00%)	2 (4.76%)

DASS-21, 21-item Depression and Anxiety Stress Scale; SF-36, 36-item short-form.

After adjusting for the stratification variables, age and gender, and the inclusion of cluster and patient ID (repeated measures) as random effects, there were no significant differences between the control and intervention patient groups on either stress (by 1.31; 95% CI −3.37 to 0.75, p=0.213, Cohen’s d=0.14), anxiety (by 0.92; 95% CI −3.77 to 1.93, p=0.525, Cohen’s d=0.10) or depression (by 0.90; 95% CI −4.23 to 2.42, p=0.595, Cohen’s d=0.08) subscales.

Descriptive analysis indicated improvements to overall mental health with time measured by the mental health component of the SF-36 V.2 for patients in the control arm and the intervention arm. However, there did not appear to be any changes in overall physical health for either the control arm or intervention arm patients (table 3).

After adjusting for the stratification variables, age and gender, and the inclusion of cluster and patient ID as random effects, there were no significant differences between the control and intervention groups on health to either physical (by 1.61; 95% CI −5.83 to 2.60, p=0.453, Cohen’s d=0.16), or mental (by 0.08; 95% CI −2.78 to 2.62, p=0.952, Cohen’s d=0.01) component summary scores.

Data related to work status by time point and arm are displayed in table 3. Eight data points referring to ‘Don’t know/Can’t say’ and ‘Refuse to say’ response options were excluded from further analysis. After adjusting for the stratification variables, age and gender, and the inclusion of cluster and repeated measures as random effects, we found no significant difference in the odds of currently working in a paid job between the control patients and the intervention patients (OR=0.82, 95% CI 0.32 to 2.13, p=0.687) for the return-to-work item.

There were a considerable number of patients (n=196) with missing observations for the DASS-21, SF-36 V.2 and work-related item at different time points. After the sensitivity analysis was conducted, results were similar to those from the non-imputed analysis (online supplemental file 3).

Due to an underpowered sample size, we are unable to report interaction effects between group and each time point. However, there was a significant effect of time on patient outcomes, with a decrease in severity of symptoms and overall mental health improving with time across three time intervals of 3 months, 6 months and 9 months. This effect was non-significant for overall physical health, nor work status, across any of the measured time intervals. This analysis is presented in online supplemental file 4.

Further sensitivity analyses were conducted for the patient outcomes adjusted for potential baseline differences. For each patient outcome, after further adjustment for baseline differences, there were no differences between the intervention and control patient groups (see online supplemental file 5).

With the purpose of expanding the existing literature, we conducted exploratory analyses to investigate whether guideline-concordant care positively impacted patient outcomes. The main patient analyses were repeated with the inclusion of 9-month GP Adherence Score as a fixed factor. After further adjustment for baseline GP Adherence Scores, increased GP Adherence Score was significantly associated with patient outcomes for decreased DASS-21 Depression Score (coefficient=−0.81, robust SE=0.39, p=0.036, 95% CI −1.57 to −0.05), and improved work status (OR=1.95, robust SE=0.41, p=0.002, 95% CI 1.29 to 2.94).

## Discussion

The IMPRovE intervention, comprising academic detailing, enrolment in a vCoP and the provision of resources, resulted in increased adherence to the Guideline recommendations. Although adherence improved for both intervention and control GPs, there was a significant increase in the adherence score for the intervention compared with control groups during this period, even after adjustment for stratification variables.

While our exploratory analysis indicates that increased adherence to the Guideline translates into a significant improvement in depression and improved work status, there was no difference in outcomes for patients attending intervention GPs compared with patients of control GPs. For all patients, there was an improvement with time in mental health symptoms. We expect all patients receiving care from a GP to improve with time due to factors such as the ongoing management of their WRMHC, and resolution of the social, health or work-related factors contributing to their condition. However, the study was underpowered to investigate potential differences between the patient outcomes between arms across the time points, with the number of recruited patients less than a third of the target sample size of 448 patients.

Patient recruitment was hampered by the COVID-19 pandemic. Participating GPs were the main source for patient recruitment, as researchers were restricted in their movements during much of the pandemic and could not do this directly due to government and employer restrictions on movement and activity. Not all GPs recruited the minimum target of seven eligible patients possibly because of the impact of the increased workload and shift to telehealth brought about by the pandemic.^20^ For patients, the combination of their WRMHC and the pressures of the pandemic may have been proven too much for them to consider participation in a research study. Our patient recruitment difficulties mirror those of other studies. The response rate in the 2021 National Return-to-work Survey conducted by Safe Work Australia declined from 67.7% in 2018 to 54.1%.^21^ Additionally, patients with mental health symptoms are traditionally hard to recruit into studies,^22^ and the topic of compensation injury adds to this difficulty as patients worry about the impact of their participation on a potential claim.

Since the GPs recruited were located throughout Australia, in urban and regional areas, and had a wide range of experience in general practice, there is confidence the findings reflect a broad range of GPs practising in Australia. There is also confidence with the veracity of the findings due to the selection of prespecified analyses. Primary analyses were performed on an intention-to-treat basis. In addition, analyses were adjusted for stratification factors (and sensitivity analysis conducted to check the robustness of the results), providing confidence that the intervention influenced adherence to Guideline recommendations.

Measurement of our primary outcome captured the diversity of patients with WRMHCs via 18 vignettes, developed based on qualitative interviews with people with lived experience of WRMHCs. The effect size for our primary analysis indicated a moderate to large effect, despite this diversity and the difficulty of adhering to guideline recommendations in such a heterogeneous patient population.

The findings of the study should be interpreted with consideration to its potential limitations. While measuring guideline adherence during consultations with patients would demonstrate a direct impact of the intervention on practice, the complexity of WRMHCs limits the ability to standardise the outcome between GPs and patients. Such an approach, especially during COVID-19, was not feasible. The use of simulations as a standardised method of assessment has been used previously to evaluate practice and as an educational tool in compensable injury research.^15 16^ However, this study-specific measure has a relatively small range of possible scores, which may have limited the change detectable from baseline to 9 months. Increasing the number of vignettes presented to GPs may have negatively impacted participant retention as participating GPs commonly have a heavy workload. The increase in GP workload during the COVID-19 pandemic made their engagement in the trial even more vulnerable. Finally, GP responses to the vignettes was a mixture of multiple choice and short answer. However, the tightened and tested scoring criteria meant that short answers were assessed for their written content, rather than their meaning being extrapolated from the often-brief responses given.

The primary outcome findings support previous research of the effectiveness of complex interventions.^7 23^ They also support the effectiveness of academic detailing and CoP for implementation of guidelines in general practice,^7^,^924 25^ and in combination with each other.^7 9^ These components of the intervention are based on flexible and trustworthy social interactions,^26^ thus potentially increasing their effectiveness to facilitate guideline implementation by: (1) Addressing professional isolation; (2) Learning from respected peers; (3) Enabling GPs to demonstrate their own leadership by assisting their peers; (4) Enabling GPs to develop confidence in their plan of action before consulting with the patient, or stakeholder involved in the patient’s care; and (5) Providing this interaction in a flexible environment that caters to the GP’s resource availability (eg, time, mode of communication).

While we have demonstrated the effectiveness of our complex intervention in improving guideline adherence, further research is needed to investigate whether other implementation approaches can achieve the same effects and the effects of these interventions on patient outcomes. Future publications from IMPRovE will describe other impacts of the intervention on other outcomes as specified in the protocol^11^ and help to contextualise the impact of the intervention with improving Guideline adherence on a wider scale. However, further research is needed to determine whether one of the active intervention components (ie, receipt of academic detailing or engagement in a vCoP) is more effective in increasing guideline-concordant practice in general practice and the applicability of these methods to guidelines addressing other clinical content areas.

## Supplementary material

10.1136/bmjment-2024-301330 online supplemental file 1

## Data Availability

Data are available upon reasonable request.
