# Supplementary material for: Implementing work-related Mental-health guidelines in general PRacticE (IMPRovE): findings of a parallel cluster randomised controlled trial
Source: BMJ Ment Health. 2025 Jul 28;28(1):e301330. doi: 10.1136/bmjment-2024-301330 (PMC12306257; doi:10.1136/bmjment-2024-301330)
Supplement: online supplemental file 1 [file bmjment-28-1-s001.pdf]

Supplementary File 1: Intervention components

Academic Detailing (AD)

AD was provided in a single 60-minute online session using video-conferencing. The session was co-facilitated by a GP opinion leader and a professional academic detailer who had been engaged through the National Prescribing Service. This is a national organisation responsible for providing evidence-based information to health professionals and consumers using interventions that have been shown to be effective to ensure the safe and efficient use of health technologies.[1] The co-facilitation of the sessions between an opinion leader and an academic detailer allowed for a seamless balance of content delivery versus structure and support throughout the session.

GP opinion leaders were GPs who had been nominated by workers' compensation authorities as these GPs were well versed in the complexities of the workers' compensation system. Each GP opinion leader was supported by an experienced academic detailer who had expertise in providing educational outreach programs to GPs. Both the GP opinion leaders and academic detailers were provided with a step-by-step guide on how to deliver the sessions and training via mock AD sessions, prior to the delivery of the AD sessions to GP-participants.

AD was organised for intervention GPs soon after randomisation and group allocation. During the session, GP-participants were detailed on how to improve care for patients with work-related mental-health conditions in accordance with the Guideline. The format of each session included 1) introduction to the Guideline and the provision of statistics about work-related mental-health conditions; 2) discussion of the key challenges that are faced by attending GPs; and 3) discussion of a case-study with advice and instruction on how to implement guideline-concordant care. Only GP-participants attending the same GP clinic attended the same AD session.

Virtual Community of Practice (vCoP)

The vCoP was an online platform that showcased the guideline recommendations and provided avenues to discuss and support guideline implementation strategies. A central component of the platform was the discussion forum. In addition, the platform offered a range of scheduled content in the form of webinars, case discussions, and news. A content roster and engagement protocol were used by the study team to plan and facilitate ongoing engagement, as monitoring engagement with pre-planned activities informed the planning of future activities. The content roster was reviewed quarterly to adjust for ongoing developments in published educational materials or other resources, and in response to GP engagement levels over the preceding quarter. After enrolment into the vCoP, GPs could access the site until the 31st of December 2022. This meant that all GPs in the intervention arm had at least 11 months access to the vCoP.

Provision of resources

Participating GPs were provided with electronic and hardcopy resources including the full guideline, flowchart of guideline recommendations, and recommended assessment tools. They were also supplied with resources from the compensation partners and Beyond Blue, a prominent non-government mental-health organisation in Australia. A hardcopy folder with printed resources was posted to the GP-participants, and an electronic version was emailed to the GPs and also available through the vCoP. Each resource included in the pack (both hardcopy and electronic versions) put together for the GPs in the intervention group, was also publicly available to any GP (including those in the control group). The difference was that the GPs receiving the intervention were provided these resources directly, rather than having to search for them online. The full guideline, short form guideline, and GP summary provided to the intervention arm GP-participants can be accessed online here: https://www.monash.edu/medicine/sphpm/general-practice/engagement/clinical-guidelines

References

1. Weekes LM, Blogg S, Jackson S, Hosking K. NPS MedicineWise: 20 years of change. J Pharm Policy Pract. 2018;11:19.

Supplementary File 2: Sensitivity analysis for secondary analysis with primary outcome

The same GPs that were missing 9-month adherence data were also missing data for the variable additional mental health training, which was used as a covariate in the secondary analysis. As this variable was ordered, the values for three GPs were able to be completed due to their being at the highest level of training (“advanced”). The remaining 18 GPs had this missing data imputed using the variables were time point, arm, cluster, state/territory, clinic size, clinic location, and gender. Ten iterations were performed. Table 1 displays the characteristics of the non-imputed and imputed additional mental health training data.

Table 1: Frequencies of additional mental health training by arm at 9-months for observed (non-imputed) and imputed values.

**Observed (Non-imputed) values**	**None**	**Basic**	**Advanced**	**Total**
Control arm				
- 9-months	2 (5·13%)	25 (64·10%)	12 (30·77%)	39 (100.00%)
Intervention arm				
- 9-months	0 (0·00%)	25 (65·79%)	13 (34·21%)	38 (100.00%)
Imputed values				
Control arm				
- 9-months	2 (1·80)	66 (59·46%)	43 (38·74%)	111 (100.00%)
Intervention arm				
- 9-months	0 (0·00%)	103 (57·54%)	76 (42·46%)	179 (100.00%)

A sensitivity analysis was conducted to check the robustness of the secondary analysis by performing multiple imputation due to missing primary outcome data. Multiple imputation of chain equations (MICE) was performed with the MICE package in Stata SE version 17.0, with details included in the Supplementary file. The sensitivity analysis was similar to the non-imputed analysis. After adjusting for the stratification variables (state, clinic size, and clinic location) and additional mental health training and inclusion of cluster as a random effect, there was a significant increase of the adherence score for the intervention group compared to controls by 0·85 (95% confidence interval: 0·30, 1·40).

Results from the sensitivity analysis were similar to those from the non-imputed analysis. Table 2 displays analyses of the non-imputed and imputed adherence scores.

After adjusting for the stratification variables (state, clinic size, and clinic location) and inclusion of cluster as a random effect, there was a significant increase of the adherence score for the intervention group compared to control by 0·95 (95% confidence interval: 0·38 - 1·53).

After adjusting for the stratification variables (state, clinic size, and clinic location) and additional mental-health training and inclusion of cluster as a random effect, there was a significant increase of the adherence score for the intervention group compared to control by 0·85 (95% confidence interval: 0·22 - 1·48).

Table 2: Mean adherence score by arm for observed (non-imputed) and imputed values

	**Arm**	**Mean adherence score**	**Median adherence score**	**SD**
Observed (Non-imputed) values	Control	5·50	5·50	1·33
	Intervention	5·82	5·50	1·67
Imputed values	Control	5·71	5·50	1·42
	Intervention	6·09	6·00	1·54

Supplementary File 3: Sensitivity analysis for secondary analysis with secondary outcomes

Variables used to impute missing DASS-21, SF-36, and work participation observations were time point, arm, cluster, state/territory, clinic size, clinic location, age, and gender. One hundred iterations were performed. Table 3 displays the characteristics of the non-imputed and imputed DASS-21 and SF-36 scores.

Table 3: Mean patient health status scores by arm for observed (non-imputed) and imputed values

	**Observed (Non-imputed) values**			**Imputed values**		
**Arm**	**Mean score**	**Median score**	**SD**	**Mean score**	**Median score**	**SD**
DASS-21 stress						
Control	21·43	20·00	9·81	19·39	19·11	9·96
Intervention	20·17	20·00	9·65	18·08	17·99	10·11
DASS-21 anxiety						
Control	14·57	14·00	9·82	12·94	12·76	9·97
Intervention	14·07	14·00	9·53	11·95	12·00	9·86
DASS-21 depression						
Control	17·41	14·00	11·98	15·56	15·21	11·40
Intervention	16·59	16·00	10·63	14·42	14·40	11·42
SF-36 physical component summary score						
Control	43·22	43·92	10·15	43·19	42·83	10·17
Intervention	42·35	43·31	10·11	42·36	42·35	10·31
SF-36 mental component summary score						
Control	33·61	33·33	11·40	36·04	36·60	11·68
Intervention	34·11	32·67	11·55	37·00	36·97	11·50
Work Participation						
	n	n		n	n	
	Yes	No		Yes	No	
Control	111	43		5136	1718	
Intervention	178	82		9881	4079	

The sensitivity analyses followed the same adjustments as the adjusted analyses of the secondary patient outcomes but using the imputed dataset. Results from the sensitivity analyses were similar to those from the non-imputed analyses.

For the DASS-21 stress scale score, control patients had non-significant greater severity of stress symptoms compared to the intervention patients by 1·24 (95% confidence interval: -3·52, 1·04; p = 0·287). There was a significant effect of time, with severity of stress symptoms decreasing with time from baseline to 3-months (-4·35; 95% confidence interval: -6·40, -2·30; p < 0·001), baseline to 6-months (-5·18; 95% confidence interval: -7·22, -3·13; p < 0·001), and baseline to 9-months (-7·86; 95% confidence interval: -10·08, -5·64; p < 0·001).

For the DASS-21 anxiety scale score, control patients had non-significant greater severity of anxiety symptoms compared to the intervention patients by 0·62 (95% confidence interval: -3·35, 2·12; p = 0·657). There was a significant effect of time, with severity of anxiety symptoms decreasing with time from baseline to 3-months (-

4·01; 95% confidence interval: -6·08, -1·94; p < 0·001), baseline to 6-months (-4·92; 95% confidence interval: -6·98, -2.86; p < 0·001), and baseline to 9-months (-6·85; 95% confidence interval: -9·24, -4·45; p < 0·001).

Note: There was an issue with convergence, so the model was altered so that patient ID was a fixed factor instead of a random factor.

For the DASS-21 depression scale score, control patients had non-significant greater severity of depression symptoms compared to the intervention patients by 0·87 (95% confidence interval: -4·03, 2·29; p = 0·590). There was a significant effect of time, with severity of depression symptoms decreasing with time from baseline to 3-months (-3·82; 95% confidence interval: -5·89, -1·75; p < 0·001), baseline to 6-months (-5·05; 95% confidence interval: -7·21, -2·88; p < 0·001), and baseline to 9-months (-7·01; 95% confidence interval: -9.57, -4.46; p < 0·001).

For the SF-36 v2 physical component summary score, control patients had non-significant better overall physical health compared to the intervention patients by 1·46 (95% confidence interval: -4·66, 1.75; p = 0·374). There was no significant effect of time on overall physical health from baseline to 3-months (-0·87; 95% confidence interval: -2·82, 1·07; p = 0·379), baseline to 6-months (-0·62; 95% confidence interval: -2·92, 1·68; p = 0·598), and baseline to 9-months (-0·26; 95% confidence interval: -2·89, 2·38; p = 0·848).

For the SF-36 v2 mental component summary score, intervention patients had non-significant better overall mental health compared to the control patients by 0·23 (95% confidence interval: -2·49, 2·95; p = 0·869). There was a significant effect of time, with overall mental health improving with time from baseline to 3-months (4·31; 95% confidence interval: 1·86, 6·76; p = 0·001), baseline to 6-months (6·53; 95% confidence interval: 3·90, 9·16; p < 0·001), and baseline to 9-months (8·90; 95% confidence interval: 5·96, 11·85; p < 0·001).

Analysis of the Return-to-Work item showed no significant difference in the odds of currently working in a paid job between the control patients and the intervention patients (Odds ratio = 0·76, 95% confidence interval: 0·37, 1·57; p = 0·458). There was also no effect of time on work status. There was no significant difference in the odds of currently working in a paid job from baseline to 3-months (Odds ratio = 1·57, 95% confidence interval: 0·84, 2·93; p = 0·153), baseline to 6-months (Odds ratio = 1·73, 95% confidence interval: 0·82, 3·66; p = 0·152), and baseline to 9-months (Odds ratio = 1·36, 95% confidence interval: 0·62, 3·00; p = 0·448).

Supplementary File 4: Effect of time point for patient outcomes

Table 4 displays the effect of time on the severity of symptoms for each subscale of the DASS-21 from baseline to each post-baseline time point.

Table 4: Confidence interval and effect size in relation to the change in symptom severity for each subscale of the DASS-21 at each time interval.

DASS-21	Time interval	Coefficient	p-value	95% confidence interval
Stress scale	baseline to 3-months	-4·32	<0·001	-6·34, -2·30
	baseline to 6-months	-4·61	<0·001	-6·58, -2·64
	baseline to 9-months	-6·96	<0·001	-9·12, -4·81
Anxiety scale	baseline to 3-months	-3·86	<0·001	-5·62, -2·10
	baseline to 6-months	-4·46	<0·001	-5·98, -2·94
	baseline to 9-months	-5·43	<0·001	-7·32, -3·53
Depression scale	baseline to 3-months	-3·57	<0·001	-5·44, -1·69
	baseline to 6-months	-4·45	<0·001	-6·10, -2·81
	baseline to 9-months	-5·81	<0·001	-7·94, -3·68

Table 5 displays the effect of time on the overall health for each component of the SF-36 at each time interval.

Table 5: Confidence interval and effect size in relation to the change in symptom severity for each component summary of the SF-36 at each time interval.

SF-36	Time interval	Coefficient	p-value	95% confidence interval
Physical component summary	baseline to 3-months	-1·42	0·023	-2·65, -0·20
	baseline to 6-months	-0·69	0·371	-2·19, 0·82
	Baseline to 9-months	0·10	0·895	-1·44, 1·65
Mental component summary	baseline to 3-months	4·47	<0·001	2·09, 6·85
	baseline to 6-months	6·42	<0·001	3·89, 8·94
	baseline to 9-months	7·48	<0·001	4·84, 10·13

There was also no effect of time on work status for any of the measured time intervals (Odds ratio from baseline to 3-months = 1·99, 95% confidence interval: 0·81, 4·89, p = 0·135; Odds ratio from baseline to 6-months = 2·51, 95% confidence interval: 0·84, 7·48, p = 0·099; Odds ratio from baseline to 9-months = 0·93, 95% confidence interval: 0·32, 2·71, p = 0·901).

Supplementary File 5: Adjustment for baseline differences in the patient outcome measures

Table 6 displays the difference between the control and intervention group patients for each of the patient outcomes when the analyses included further adjustment for baseline differences.

Table 6: Differences between control and intervention group patients for patient outcomes when further adjusted for baseline outcome differences

	**Coefficient**	**p-value**	**95% confidence interval**
DASS-21 Stress scale	-1·80	0·295	-5·16, 1·57
DASS-21 Anxiety scale	-2·56	0·083	-5·45, 0·33
DASS-21 Depression scale	-2·35	0·185	-5·82, 1·12
SF-36 physical component score	0·37	0·678	-1·40, 2·15
SF-36 mental component score	1·15	0·535	-2·47, 4·76
	**OR**	**p-value**	**95% confidence interval**
Return to work item	2·20	0·226	0·61, 7·92
